# Adding Maximum Standard Uptake Value of Primary Lesion and Lymph Nodes in ^18^F-Fluorodeoxyglucose PET Helps Predict Distant Metastasis in Patients with Nasopharyngeal Carcinoma

**DOI:** 10.1371/journal.pone.0103153

**Published:** 2014-07-28

**Authors:** Qi Shi, Zhongyi Yang, Yingjian Zhang, Chaosu Hu

**Affiliations:** 1 Department of Radiation Oncology, Fudan University Shanghai Cancer Center, Department of Oncology, Shanghai Medical College, Fudan University, Shanghai, China; 2 Department of Nuclear Medicine, Fudan University Shanghai Cancer Center, Department of Oncology, Shanghai Medical College, Fudan University, Shanghai, China; University of California Davis, United States of America

## Abstract

**Objective:**

To find out the most valuable parameter of ^18^F-Fluorodeoxyglucose positron emission tomography for predicting distant metastasis in nasopharyngeal carcinoma.

**Methods:**

From June 2007 through December 2010, 43 non-metastatic NPC patients who underwent ^18^F-Fluorodeoxyglucose positron emission tomography/computed tomography (PET/CT) before radical Intensity-Modulated Radiation Therapy were enrolled and reviewed retrospectively. PET parameters including maximum standardized uptake value (SUVmax), mean standardized uptake value (SUVmean), metabolic tumor volume (MTV), and total lesion glucose (TLG) of both primary tumor and cervical lymph nodes were calculated. Total SUVmax were recorded as the sum of SUVmax of primary tumor and cervical lymph nodes. Total SUVmean, Total MTV and Total TLG were calculated in the same way as Total SUVmax.

**Results:**

The median follow-up was 32 months (range, 23–68 months). Distant metastasis was the main pattern of treatment failure. Univariate analysis showed higher SUVmax, SUVmean, MTV, and TLG of primary tumor, Total SUVmax, Total MTV, Total TLG, and stage T3-4 were factors predicting for significantly poorer distant metastasis-free survival (p = 0.042, p = 0.008, p = 0.023, p = 0.023, p = 0.024, p = 0.033, p = 0.016, p = 0.015). In multivariate analysis, Total SUVmax was the independent predictive factor for distant metastasis (p = 0.046). Spearman Rank correlation analysis showed mediate to strong correlationship between Total SUVmax and SUVmax-T, and between Total SUVmax and SUVmax-N(Spearman coefficient:0.568 and 0.834;p = 0.000 and p = 0.000).

**Conclusions:**

Preliminary results indicated that Total SUVmax was an independently predictive factor for distant metastasis in patients of nasopharyngeal carcinoma treated with Intensity-Modulated Radiation Therapy.

## Introduction

Nasopharyngeal carcinoma (NPC) is the one of the most endemic head and neck cancer in Southeast Asia and East Asia. The radiotherapy has been used as a treatment of the choice for NPC since the middle of the last century. With the advancing of the radiation technologies, such as Intensive-Modulated Radiation Therapy (IMRT), and concurrent chemoradiotherapy, the locoregional control and survival of NPC[1], [2] have improved significantly. It has also reduced the normal tissue toxicities as well. However, the distant metastasis is the main obstacle for further improving the treatment outcomes [3]–[5].

To date, the AJCC (American Joint Committee on Cancer) staging system has been considered as the most important prognostic factor [6]–[8]. However, this system is mainly based on the anatomic imaging studies and clinical physical examines. With the new diagnostic tools available, clinicians are looking for more prognostic factors which predict treatment outcome more accurately and guide the individualized treatments, and thus improve the prognosis.

^18^F-Fluorodeoxyglucose positron emission tomography (^18^F-FDG PET) has been wildly used in the initial diagnosis and staging workup for newly diagnosed malignancies, including NPC patients. It is still questionable that whether ^18^F-FDG PET is more valuable in detecting the primary tumor and cervical lymph nodes metastases of head and neck cancer comparing with computed tomography (CT) or other imaging studies [9]–[13]. A few studies have showed the higher sensitivity and specificity in regarding the detecting of distant metastasis [14]–[18].

As a functional imaging study, ^18^F-FDG PET may have a predictive value in long-term prognosis. The studies [19]–[21] have found the prognostic value of ^18^F-FDG PET for head and neck cancer. Researches of its prognostic value for nasopharyngeal carcinoma have also been conducted recently. The relationship between SUVmax (maximum standardized uptake value) and prognosis of NPC is most interested. Some studies have suggested that higher uptake value is correlated with poorer DMFS (distant metastasis-free survival), DFS (disease-free survival) and OS (overall survival) [22]–[25]. In addition, MTV (metabolic tumor volume) and TLG (total lesion glucose) of primary tumor were observed to be negatively correlated with DFS and OS [26]–[28].

^18^F-FDG PET can provide more functional information by various parameters. The most clinical studies have only utilized the traditional factors such as SUVmax and MTV. In order to get the more benefits from the ^18^F-FDG PET, we measured SUVmax, SUVmean (mean standardized uptake value), MTV and TLG of primary tumor and metastatic lymph nodes. Total SUVmax, Total SUVmean, Total MTV and Total TLG, which represented the overall metabolic activity of both primary lesion and lymph nodes, were also been calculated.

## Materials and Methods

### Ethics Statement

This study was approved by the Research Ethics Committee of Fudan University Shanghai Cancer Center and was performed in accordance with the ethical standards laid down in the 1964 Declaration of Helsinki and all subsequent revisions. All patients provided their written informed consent to participate in the study.

### Patients and pretreatment evaluation

From June 2007 to December 2010, 43 patients with NPC diagnosed and treated in our hospital were enrolled and reviewed retrospectively. The inclusion criteria were as following: (1) biopsy-proven primary nasopharyngeal carcinoma; (2) with pretreatment magnetic resonance imaging (MRI) of the nasopharynx and neck, and whole-body ^18^F-FDG PET/CT; (3) receiving radiotherapy in our institute; (4) the age was from 18 to 70 years old; (5) with completed medical history. The exclusion criteria were as follows: (1) with distant metastasis proven by clinical or radiologic evidence; (2) previously radiotherapy, chemotherapy or surgery of other anti-cancer treatment before undergoing whole-body ^18^F-FDG PET/CT; (3) with any other primary malignant tumor previously diagnosed, or combined concurrently; (4) with diabetes mellitus. All patients were staged according to 2010 UICC/AJCC (Union for International Cancer Control/American Joint Committee on Cancer) staging system.

Pretreatment evaluation were composed of complete medical history, physical examination, indirect or fiberoptic endoscopic examination of nasopharynx, biopsy of the neoplasm in nasopharynx, MRI scans of nasopharynx and neck, and whole-body ^18^F-FDG PET/CT.

### PET/CT imaging and imaging interpretation

^18^F-FDG was produced by cyclotron (Siemens CTI RDS Eclips ST, Knoxville, Tennessee, USA) using Explora FDG4 module in our center. Radiochemical purity was over 95%.

All the patients were requested to fast at least 4 h before ^18^F-FDG PET/CT scan. At the time of the tracer injection (dosage: 7.4MBq/kg), patients' serum glucose should be controlled between 3.9 mmol/L and 7.1 mmol/L. Before and after injection, patients were kept lying comfortably in a quiet, dimly lit room. Scanning was initiated 1 h after administration of the tracer. The images were obtained on a Siemens biograph 16HR PET/CT scanner (Knoxville, Tennessee, USA). The transaxial intrinsic spatial resolution was 4.1 mm (full-width at half-maximum) in the center of the field of view. The data acquisition procedure was as follows: CT scanning was first performed, from the proximal thighs to head, with 120 kV, 80∼250 mA, pitch 3.6, rotation time 0.5. Immediately after CT scanning, a PET emission scan that covered the identical transverse field of view was obtained. Acquisition time was 2∼3 min per table position. PET image data sets were reconstructed iteratively by applying the CT data for attenuation correction, and coregistered images were displayed on a workstation.

A multimodality computer platform (Syngo, Siemens, Knoxville, Tennessee, USA) was used for image review and manipulation. Two experienced nuclear medicine physician evaluated the images independently. The reviewers reached a consensus in cases of discrepancy.

Quantification of glucose metabolic activity was obtained using the Standardized Uptake Value (SUV) normalized to body weight. The maximum and mean SUV for primary tumor (SUVmax-T, SUVmean-T) and neck lymph nodes (SUVmax-N, SUVmean-N) were calculated. Besides, MTV was also recorded. The boundaries were drawn large enough to include the primary tumor within the nasopharynx or lymph nodes in the axial, coronal, and sagittal PET images. To define the contouring margins around the target, we used an SUV of 2.5 based on the experience of previous investigators [26], [27], [29], [30]. The contour around the target lesion inside the boundaries was automatically produced and the voxels presenting SUV intensity of greater than 2.5 within the contouring margin were incorporated to define the MTV. TLG was calculated according to the following formula: TLG  =  SUVmean×MTV. MTV and TLG of primary tumor were recorded as MTV-T, TLG-T, and those of the sum of all neck lymph nodes were recorded as MTV-N, TLG-N. Total SUVmax were recorded as the sum of SUVmax of primary tumor and cervical lymph nodes. Total SUVmean, Total MTV and Total TLG were calculated in the same way as Total SUVmax. Parameters of retropharyngeal lymph nodes were calculated as those of cervical lymph nodes.

### Radiotherapy

#### Intensity-Modulated Radiation Therapy (IMRT)

Patients were immobilized in the supine position with a thermoplastic mask. CT simulation was performed after immobilization, obtaining 3-mm slices from the anterior clinoid process to the hyoid bone, and 5-mm slices from the hyoid bone to 2 cm below the sternoclavicular joint. The gross tumor volume (GTV) included primary tumor and enlarged RLNs. The clinical target volume (CTV) included bilateral coverage of levels II, III, VA and RLNs. The CTV should also cover the entire nasopharynx, parapharyngeal space, clivus, base of skull, pterygoid fossa, posterior half of ethmoidal sinus, inferior sphenoid sinus, and posterior third of nasal cavity and maxillary sinuses. A margin of 3–5 mm around GTV and CTV should be added to account for the patient motion and set-up error. The total dose to primary tumor was 66 Gy in 30 fractions for T1 or T2 disease, 70.4 Gy in 32 fractions for T3 or T4 lesion. A total dose of 60 Gy was delivered to the CTV in 30–32 fractions.

#### Boost

Residual disease diagnosed by clinical examination or MRI was boosted. Brachytherapy was used for superficial lesion, with a dose of 6–16 Gy in one or two weekly fractions. More advanced disease was boosted by small field, including IMRT, 3D-RT, 2D-RT and stereotactic radio-surgery and the dose was 6–8 Gy.

### Chemotherapy

There were several regimens of neoadjuvant chemotherapy and adjuvant chemotherapy in our institution, including PF, TPF and GP. The TPF protocol consisted of docetaxel 75 mg/m^2^ IV on day 1, cisplatin75 mg/m^2^ IV on day 1, and 5-fu 500 mg/m^2^ d continuously IV on day1–5. The PF protocol consisted of cisplatin 75 mg/m^2^ IV on day 1 and 5-fu 500 mg/m^2^ d continuously IV on day 1–5. The GP regimen included cisplatin 75 mg/m^2^d IV on day 1 and gemcitabine 1000 mg/m^2^ IV on day 1, 8. Regimens were repeated every 3 weeks for 2 cycles as neoadjuvant chemotherapy. This was followed by cisplatin 40 mg/m^2^ IV weekly during radiation. Adjuvant chemotherapy were repeated every 3–4 week for 3 cycles.

### Follow-up

The follow-up period was from the first day of initiation of treatment until death or last visit. The median follow-up period was 32 months (range, 23–68 months).

Patients were followed every 3 months in the first two year, then every 6 months in the following three years and then, once a year thereafter. In each visit, medical history was collected, physical examinations including nasopharyngoscopy were performed. Nasopharyngeal MRI was performed 3 months and 1 year after completion of radiotherapy, and every 6 months in the second to fifth year, and then yearly thereafter. The following tests were done at least once a year: chest CT or X-ray, abdominal sonography, and bone scan when clinically indicated. Late toxicities were evaluated according to the toxicity criteria of the RTOG [6].

### Statistical analysis

All analyses were performed by using the SPSS software, version 19.0. The actuarial rates were estimated by the Kaplan–Meier method [31]. The primary endpoint was DMFS, and the secondary endpoints were PFS (progression-free survival) and OS. All the endpoints were defined as the interval from the date of initiation of treatment to the date of the failure or death, or last follow-up. Disease progression was defined as the developing of local or regional recurrence, or distant metastasis. The parameters of FDG PET used in statistical analysis included: SUVmax-T, SUVmean-T, MTV-T, TLG-T, SUVmax-N, Total SUVmax, Total SUVmean, Total MTV and Total TLG. The best cut-off values of SUVmax, SUVmean, MTV and TLG, which showed the best trade-off between sensitivity and specificity for DMFS, were determined by receiver operating characteristic (ROC) analysis following the method of Metz [32].The survival curves were compared with the log-rank test. Independent sample T test was used to test whether there were significant differences in the value of above PET parameters between patients of different clinical stages, and Mann-Whitney test was used when heterogeneity of variance existed. Multivariate analyses with the Cox proportional hazards model [33] were used to test for independent significance by backward elimination of insignificant explanatory variables of the different parameters.

## Results

### Patient characteristics and treatment outcome

There were 43 patients enrolled in this study. The patient characteristics are listed in Table 1. At the time of this study, 4 patients had died and 39 were alive, 4 experienced local recurrence and 2 experienced nodal recurrence, and distant metastases occurred in 8 patients (Table 2). Distant metastasis is the main treatment failure, and the common sites of distant failure were liver, lung and bone. The 3-year DMFS, PFS, and OS were 81.0%,69.1% and 83.2%, respectively.

**Table 1:** Patient characteristics.

	Number of patients	%
Age		
Median	45 (yeas)	
Range	18–69 (years)	
Sex		
Male	32	74.42
Female	11	25.58
Pathology		
WHO II	8	18.60
WHO III	35	81.40
AJCC stage		
I-II	11	25.58
III-IV	32	74.42
T stage		
T1-2	22	51.16
T3-4	21	48.84
N stage		
N0-1	17	39.53
N2-3	26	60.47
Concurrent chemotherapy		
Yes	19	44.19
No	24	55.81
Patterns of chemotherapy		
None	6	13.95
NAC	6	13.95
NAC+AC	12	27.91
NAC+CCRT	19	44.19

Abbreviation: NAC, neoadjuvant chemotherapy; AC, adjuvant chemotherapy; CCRT, concurrent chemoradiotherapy.

**Table 2:** Patterns of failure.

Failure	Number of patients	%
Local recurence	2	4.65
Nodal recurrence	2	4.65
Distant metastasis	6	13.95
Local recurrence & distant metastasis	2	4.65
Death	4	9.30

### Differences in PET parameters between patients with different characteristics

**Figure 1:**
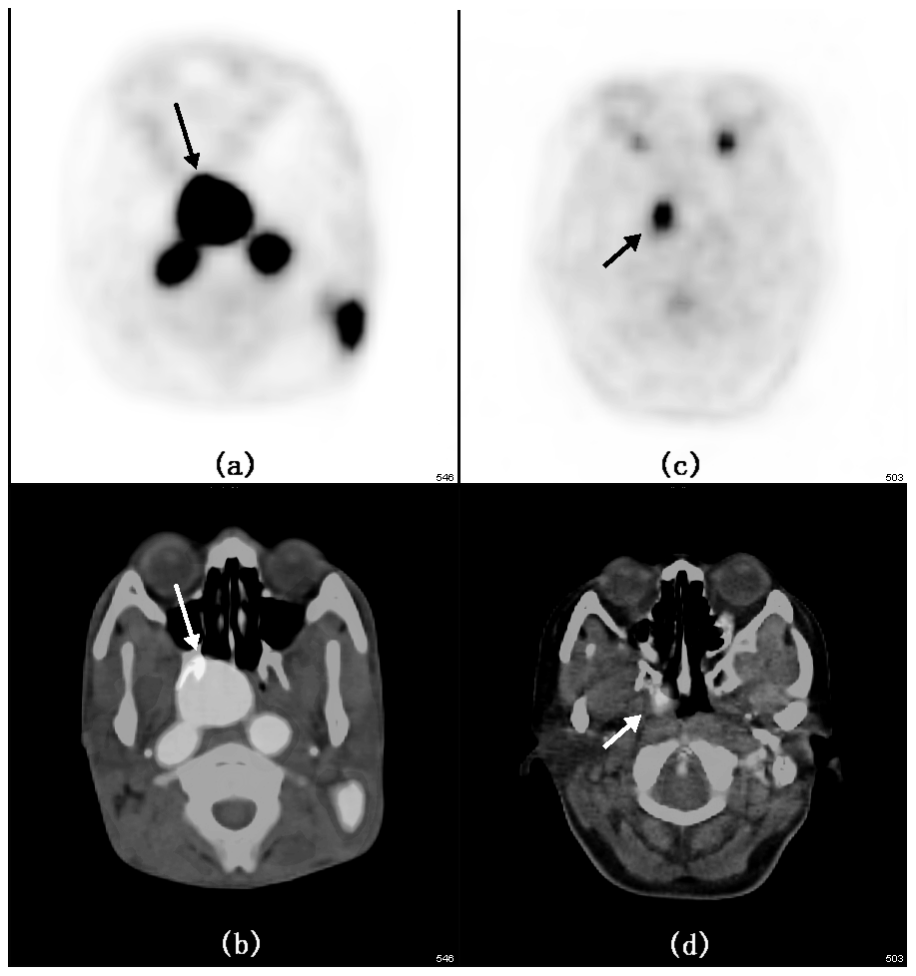
(a) and (b) were PET image and PET/CT infusion image respectively, showing the primary lesion of a patient with T3N1M0 disease, with SUVmax-T (maximal standardized uptake value of primary tumor) of 20.30 and Total SUVmax (sum of maximal standardized uptake value of primary tumor and cervical lymph nodes) of 58.73. This patient developed liver metastasis 8 months after treatment. (c) and (d) were PET image and PET/CT infusion image respectively, showing the primary lesion of a patient with T1N0M0 disease, with both SUVmax-T and Total SUVmax of 6.71. Neither recurrence nor distant metastasis has happened in this patient till now. The arrows pointed after the primary lesions with increasing uptake of FDG.

MTV-T, TLG-T, Total SUVmax, Total SUVmean, Total MTV and Total TLG of patients with stage III-IVB (according to 2010 AJCC staging system) diseases were significantly higher than those with stage I-II diseases (p value were 0.001, 0.001, 0.022, 0.008, 0.000 and 0.016, respectively). MTV-T, SUVmax-N, Total SUVmax, Total SUVmean, Total MTV and Total TLG of patients with N2-3 diseases were also higher than those with N0-1 diseases (p = 0.029, p = 0.002, p = 0.000, p = 0.000, p = 0.000 and p = 0.002, respectively). Patients with T3-4 diseases showed higher MTV-T, TLG-T and Total MTV than those with T1-2 diseases (p = 0.000, p = 0.000 and p = 0.007, respectively). There was no difference was observed in PET parameters among sex, age or pathology. Figure 1 showed the PET/CT images of two different patients.

### Cut-off values of PET parameters

As DMFS was the primary endpoint, we took the values of PET parameters showing the best trade-off between sensitivity and specificity for DMFS as the best cut-off values, which were determined by receiver operating characteristic (ROC) analysis. The best cut-off values of SUVmax-T, SUVmean-T, MTV-T, TLG-T, SUVmax-N, MTV-N, TLG-N, Total SUVmax, Total SUVmean, Total MTV, Total TLG were 8.70, 4.30, 12.71, 58.08, 6.40, 27.14, 16.04, 49.79 and 235.46, respectively.

### Univariate analysis of factors for survival

**Figure 2:**
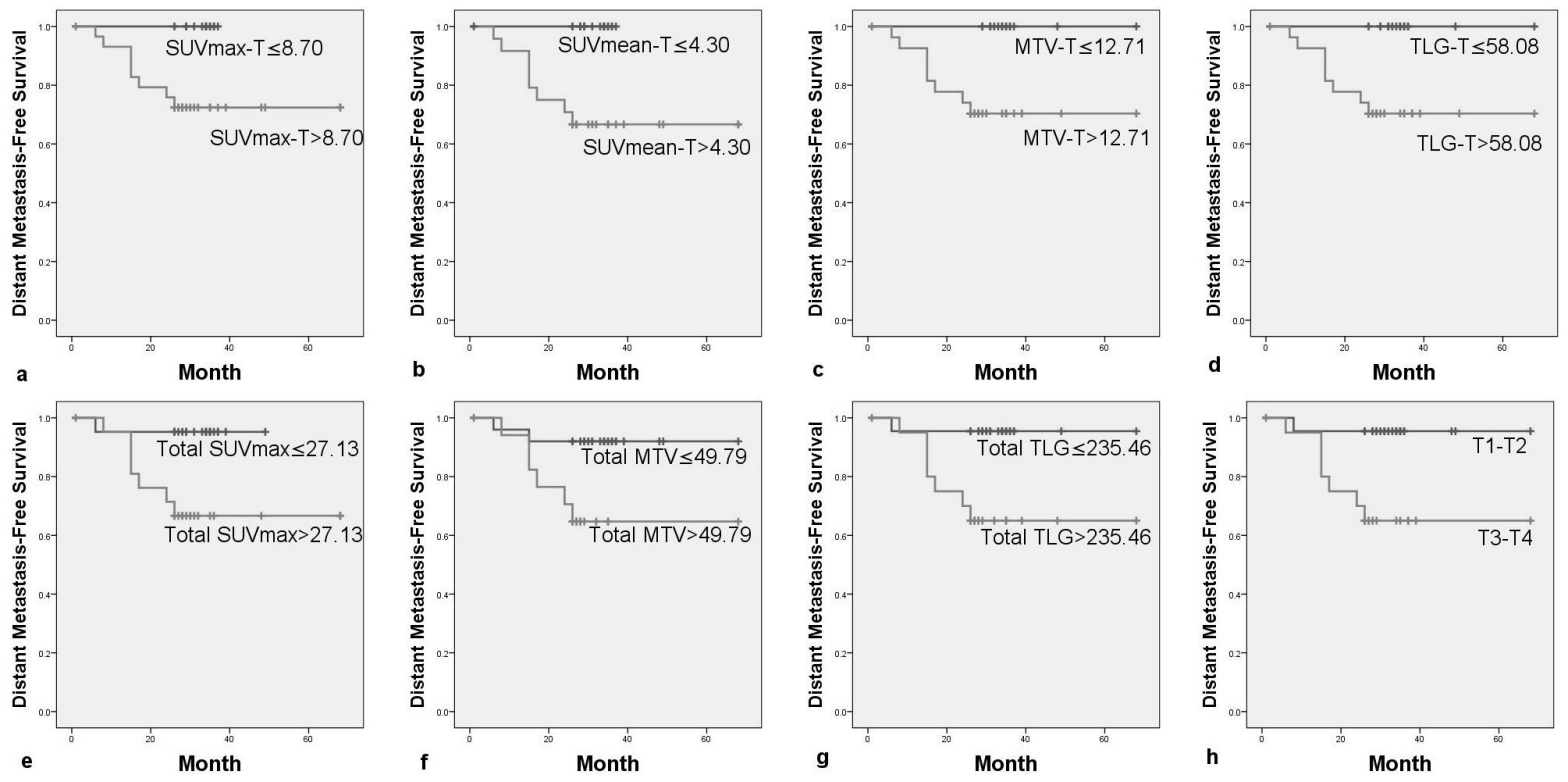
Distant metastasis-free survival by: (a) maximal standardized uptake value of primary tumors (SUVmax-T), (b) mean standardized uptake value of primary tumors (SUVmean-T), (c) metabolic tumor volume of primary tumors (MTV-T), (d) total lesion glucose of primary tumors (TLG-T), (e) sum of maximal standardized uptake value of primary tumors and cervical lymph nodes (Total SUVmax), (f) sum of metabolic tumor volume of primary tumors and cervical lymph nodes (Total MTV), (g) sum of total lesion glucose of primary tumors and cervical lymph nodes (Total TLG), (h) T stage of primary tumors.

The summarized results of univariate analysis were listed in Table 3. Patients met any of the following factors including lower SUVmax-T (SUVmax-T≤8.70), SUVmean-T (SUVmean-T≤4.30), MTV-T (MTV-T≤12.71), TLG-T (TLG-T≤58.08), Total SUVmax (Total SUVmax≤27.14), Total MTV (Total MTV≤49.79) or Total TLG (Total TLG≤235.46) had a better DMFS statistically (p = 0.042, p = 0.008, p = 0.023, p = 0.023, p = 0.024, p = 0.033, p = 0.016)(Figure 2). Patients with a lower Total SUVmean (Total SUVmean≤16.04) only showed a trend of better DMFS (p = 0.074). Those with SUVmean-T≤4.30 had a better PFS (p = 0.027), but patients with Total TLG≤235.46 were observed only a trend of better PFS (p = 0.084). In addition, patients with a lower SUVmean-T (SUVmean-T≤4.30) or MTV-T (MTV-T≤12.71) had a relatively higher OS (p = 0.079,p = 0.073). Those with a lower SUVmax-N (SUVmax-N≤6.40) showed no change in DMFS, PFS and OS. Among other clinical and pathological factors including sex, age (age>41.5 vs age≤41.5), T stage, N stage, overall stage, receiving concurrent chemoradiotherapy or not, and patterns of chemoradiotherapy, only patients with T1-2 diseases had a better DMFS (p = 0.015) and OS (p = 0.035) than T3-4 patients, and showed a trend of better PFS (p = 0.085).

**Table 3:** Univariate analysis of PET parameters and other clinical factors for 3-year DMFS, PFS and OS.

Variable		Number of patients	3-year DMFS(%)	P	3-year PFS(%)	P	3-year OS(%)	P
Age	≤45	22	77.3	0.491	67.5	0.574	89.9	0.871
	>45	21	85.0		64.8		73.3	
Sex	Male	32	80.6	0.880	78.1	0.192	76.5	0.830
	Female	11	81.8		41.6		90.9	
AJCC stage	I-II	11	90.9	0.367	72.7	0.986	100	0.221
	III-IVB	32	77.4		67.6		77.3	
	T1-T2	22	95.5	***0.015***	81.3	0.085	100	***0.035***
	T3-T4	21	65		55.6		67.9	
	N0-N1	17	82.4	0.949	70.6	0.720	85.9	0.657
	N2-N3	26	80		67.8		78.7	
Concurrent chemotherapy	Yes	19	78.9	0.745	61.4	0.598	88.4	0.810
	No	24	82.6		74.2		78.4	
Patterns of chemotherapy	None	6	100	0.194	100	0.147	100	0.533
	NAC	6	100		62.5		62.5	
	NAC+AC	12	63.6		66.7		66.7	
	NAC+CCRT	19	78.9		76.7		73.7	
Pathology	WHO II	8	75	0.657	46.9	0.293	65.6	0.202
	WHO III	35	82.4		73.3		92.2	
SUVmax-T	≤8.69	13	100	***0.042***	84.6	0.205	100	0.151
	>8.69	30	72.4		60.6		75.9	
SUVmean-T	≤4.29	19	100	***0.008***	89.5	***0.027***	100	0.079
	>4.29	24	66.7		53.8		73.5	
MTV-T	≤12.70	15	100	***0.023***	70	0.472	100	0.073
	>12.70	28	70.4		71.4		70.6	
TLG-T	≤58.08	15	100	***0.023***	79.4	0.218	100	0.098
	>58.08	28	70.4		62.5		73.2	
SUVmax-N	≤6.39	27	88.5	0.117	74.1	0.252	91.9	0.570
	>6.39	16	68.8		61.1		68.2	
Total SUVmax	≤27.13	21	95.2	***0.024***	77.1	0.182	94.1	0.331
	>27.13	22	66.7		62.9		73.7	
Total SUVmean	≤16.04	24	91.3	0.074	75.8	0.224	91.0	0.768
	>16.04	19	68.4		61.6		73.3	
Total MTV	≤49.79	25	92.0	***0.033***	72.1	0.299	91.4	0.531
	>49.79	18	64.7		66.7		60.0	
Total TLG	≤235.46	22	95.5	***0.016***	77.7	0.084	94.7	0.221
	>235.46	21	65.0		61.1		69.7	

Bold numbers, indicated there was statistically significant difference (p value <0.05).

### Multivariate analysis of independent factors for survival

Factors including SUVmax-T, SUVmean-T, MTV-T, TLG-T, Total SUVmax, Total MTV, Total TLG, T stage, which were proved by univariate analysis to have the potential affecting DMFS were included in the Cox proportional hazard model. Hazard ratio (represented by EXP(B) in Table 4) of SUVmean-T, MTV-T, Total SUVmax and Total TLG were larger than 1. But only Total SUVmax had a p value less than 0.05 (p = 0.046). Thus, the result indicated that Total SUVmax is the independently predictive factor for DMFS (Hazard ratio: 2.623, Confidence interval of hazard ratio: 1.018 - 6.755, p = 0.046) (Table 4). Similarly, SUVmean-T, the potential factor proved by univariate analysis, and Total TLG and T stage, which led to trends of changes in PFS were included into the Cox proportional hazard model, and SUVmean-T were showed to be the independently predictive factor for PFS (Hazard Ratio: 1.499, Confidence interval of hazard ratio: 1.049 – 2.144, p = 0.026) (Table 4). Factors leading to trends of differences in OS including SUVmean-T, MTV-T and T stage were also included into the COX model, and the result indicated SUVmean-T to be the independent factor for OS (Hazard Ratio: 2.028, Confidence interval of hazard ratio: 1.052 – 3.911, p = 0.035) (Table 4).

**Table 4:** Summary of multivariate analysis.

Endpoint	Variable	*B	P value	#EXP(B)	95% CI for EXP(B)
DMFS	SUVmax-T	−9.221	0.055	0.000	0.000–1.196
	SUVmean-T	59.839	0.055	9.725E25	0.301–3.142E52
	MTV-T	3.946	0.061	51.703	0.840–3182.561
	TLG-T	−0.861	0.61	0.423	0.172–1.039
	Total SUVmax	0.964	***0.046***	2.623	1.018–6.755
	Total MTV	−1.118	0.065	0.327	0.100–1.072
	Total TLG	0.187	0.066	1.206	0.988–1.473
	T stage	−164.227	0.059	0.000	0.000–368.385
PFS	SUVmean-T	0.405	***0.026***	1.499	1.049–2.144
	Total TLG	0.000	0.891	1.000	0.997–1.002
	T stage	−1.854	0.089	0.157	0.019–1.326
OS	SUVmean-T	0.707	***0.035***	2.028	1.052–3.911
	MTV-T	−0.022	0.495	0.979	0.919–1.042
	T stage	−11.991	0.952	0.000	0.000–1.30E163

^*^ B represented the regression coefficient for each variable in Cox model.

# EXP(B) represented the hazard ratio (HR) of each variable.

Bold numbers, indicated there was statistically significant difference (p value <0.05).

Abbreviation: CI, Confidence Interval.

### Spearman Rank correlation analysis

Total SUVmax showed moderate to strong correlation with SUVmax-T and SUVmax-N (Spearman Rank coefficient:0.568 and 0.834, respectively;p = 0.000 and p = 0.000, respectively). But it only had mild correlation with stage of disease (Spearman Rank coefficient: 0.399, p = 0.008). There was especially strong correlationship between SUVmean-T and SUVmax-T (Pearson coefficient:0.905,p = 0.000), and moderate to strong correlationship was observed beween SUVmean-T and MTV-T, or between SUVmean-T and TLG-T (Pearson coefficient:0.474 and 0.621, respectively;p = 0.001 and p = 0.000, respectively).

## Discussion

The prognostic factors for malignant tumor have always been attracting a lot of attention, for their potential of guiding the clinicians to conduct personalized treatments for every patients. ^18^F-FDG PET, as a functional imaging, is promising to provide the bio-metabolic information of the tumor to clinical doctors. The FDG PET is considered as a non-invasive functional imaging study. It could provide more biological characters of tumors, such as cell viability, proliferative activity, hypoxia, low apoptosis rate, and p53 over-expression [34].

Our study has utilized the metabolic information of FDG PET, and tried to find the most valuable parameters for treatment outcome of nasopharyngeal carcinoma from SUVmax-T, SUVmean-T, MTV-T and TLG-T of primary tumor, SUVmax-N of metastatic lymph nodes, and Total SUVmax, Total SUVmean, Total MTV and Total TLG.

There were several studies focusing on the predictive value of FDG PET for distant metastasis of NPC after treatment. These studies limited by analyzing no more than two PET parameters, and always focused on traditional parameters. Among the parameters, SUVmax received the most attention. Lee analyzed 41 patients of NPC who had received radical radiotherapy and found that those with lower SUVmax had better DFS(disease-free survival) [22]. The research conducted by Xie also indicated a similar conclusion that the higher the SUVmax of primary tumor, the lower the DFS and OS [24]. However, the two studies failed to point out the definite pattern of treatment failure (for example, local recurrence or distant metastasis). These studies were limited by unable to guide treatments. Chan and Hung conducted further studies and found that SUVmax was correlated with DMFS. The results of Chan indicated that the 5-year DMFS in stage IVa–b patients with SUVmax >12.0 was significantly lower than that in stage I–III patients with SUVmax≤12 (p = 0.0001) [23]. The conclusion of Hung showed that both SUVmax of the primary tumor and that of neck lymph nodes were independent prognostic factors for DMFS in NPC patients treated with IMRT [25]. Nevertheless, our study revealed that SUVmax-T, SUVmean-T, MTV-T, TLG-T, Total SUVmax, Total MTV, Total TLG and T stage were all proved to be potential factors negatively affected the 3-year DMFS in univariate analysis, after included into Cox proportional hazards model, only Total SUVmax was the independently prognostic factor for DMFS. Moreover, in Spearman Rank correlation analysis, Total SUVmax, as a parameter reflecting total tumor burdon, showed moderate to strong correlation with SUVmax-T and SUVmax-N. So it is reasonable to hypothesize that Total SUVmax was the parameter most directly correlated with DMFS. As none of previous studies included Total SUVmax into analysis, they finally drew conclusion that SUVmax of primary tumor or cervical lymph nodes was the most valuable predictive parameter of patients with NPC, and might have been insufficient. The result of our study indicates that patients with higher Total SUVmax may have higher risk of developing distant metastasis, and need more aggressive systematic treatment.

The multivariate analysis from this study also discovered that mean standardized uptake value of primary tumor (SUVmean-T) was independent factor for PFS and OS. However, this result should be interpreted with caution, as all of optimal cut-off values of PET parameters by ROC analysis were based on the best trade-off between sensitivity and specificity for DMFS, not for PFS or OS.

In our study, all patients were treated with IMRT, and half of the patients received concurrent chemoradiotherapy. The distant metastasis remained the main pattern of treatment failure. It confirmed the previous studies that IMRT and concurrent chemotherapy have greatly improved the local-regional control of NPC, without a major impact on distant metastasis [3]-[5]. There was a randomized phase II trial reported the results in stage III-IVB NPC patients, comparing CCRT with or without neoadjuvant docetaxel and cisplatin. This clinical trial confirmed that the neoadjuvant chemotherapy is beneficial for improving DMFS and OS [35]. Meanwhile, there are several studies having similar conclusion. The treatment related toxicities were tolerable [36], [37]. In addition to chemotherapy, biological target therapy is newer type of systematic treatment. The Radiation Therapy Oncology Group have conducted a study of concurrent chemoradiotherapy followed by adjuvant chemotherapy with bevacizumab (RTOG 0615). The results indicated that the therapy is feasible and may delay the progression of subclinical distant disease [38]. Another phase II clinical trial of cetuximab with concurrent chemoradiotherapy in locoregionally advanced NPC also showed that the strategy is feasible. The preliminary rate was favorable comparing with historic data [39]. It is our opinion that should the patients with high risk of distant metastasis be predicted by Total SUVmax of FDG PET, more aggressive systematic treatment could be considered in these patients.

There are still limitations in this study. As it is a retrospective study, there exist defects in data completeness and comparability. The total amount of patients enrolled in was relatively small. With the accumulation of patients and follow-up period, we will conduct further analysis with more patients and longer follow-up period on the basis of the current preliminary results. It is possible that we may find more valuable parameters of FDG PET for the predicting the prognosis of NPC patients, and thus help to guide personalizing treatment of NPC better.

In conclusion, the preliminary results of our study showed that Total SUVmax was an independently predictive factor for distant metastasis in NPC patients. This may assist the clinicians to conduct personalized treatment that is more aggressive in these patients.
